# High expression of serine protease, *Brachyurin* in the posterior midgut of black soldier fly (*Hermetia illucens*) during horse dropping processing

**DOI:** 10.1186/s13104-024-06846-0

**Published:** 2024-06-29

**Authors:** Megumi Wakuda, Takuma Sakamoto, Akane Tanaka, Satoshi Sugimura, Yuki Higashiura, Takeru Nakazato, Hidemasa Bono, Hiroko Tabunoki

**Affiliations:** 1 Department of Science of Biological Production, Graduate School of Agriculture, Tokyo University of Agriculture and Technology, Fuchu, Tokyo Japan; 2 Division of Animal Life Science, Institute of Agriculture, Tokyo, Japan; 3 Laboratory of Comparative Animal Medicine, Division of Animal Life Science, Institute of Agriculture, Tokyo University of Agriculture and Technology, Fuchu, Tokyo Japan; 4 Cooperative Major in Advanced Health Science, Graduate School of Bio-Applications and System Engineering, Tokyo University of Agriculture and Technology, Fuchu, 183-8509 Tokyo Japan; 5 Institute of Global Innovation Research, Tokyo University of Agriculture and Technology, Fuchu, 183-8509 Tokyo Japan; 6 Database Center for Life Science (DBCLS), Joint Support-Center for Data Science Research, Research Organization of Information and Systems (ROIS), Mishima, 411-8540 Shizuoka Japan; 7 Laboratory of Bio-DX, Genome Editing Innovation Center, Hiroshima University, Higashi-Hiroshima city, Hiroshima, 739-0046 Japan; 8 Laboratory of Genome Informatics, Graduate School of Integrated Sciences for Life, Hiroshima University, Higashi-Hiroshima city, Hiroshima, 739-0046 Japan

**Keywords:** Black soldier fly (BSF), *H. Illucens*, Livestock, Feed, Serine protease, Digestive enzyme, Dropping processing

## Abstract

**Objective:**

Livestock droppings cause some environmental problems, but they have the potential to be used as effective biomass resources. The black soldier fly (BSF), *Hermetia illucens* (Diptera: Stratiomyidae), is suitable for efficiently processing such resources. By using BSF larvae for the disposal of livestock droppings, we can obtain two valuable products: protein resources and organic fertilizer. However, there is insufficient research on the digestive enzymes suitable for processing this waste. Here, we aimed to construct an efficient BSF processing system using livestock droppings, and we explored the digestive enzymes involved in this process.

**Results:**

First, we investigated the characteristics of transcripts expressed in the midgut of BSF larvae and found that immune response-related genes were expressed in the midgut. Then, we investigated digestive enzymes and identified a novel serine protease, *HiBrachyurin*, whose mRNA was highly expressed in the posterior midgut when BSF larvae fed on horse droppings. Despite the low protein content of horse droppings, larvae that fed on horse droppings accumulated more protein than those in the other groups. Therefore, *HiBrachyurin* may contribute to digestibility in the early stage of protein degradation in BSF larvae fed on horse droppings.

**Supplementary Information:**

The online version contains supplementary material available at 10.1186/s13104-024-06846-0.

## Introduction

Increasing livestock production in response to the constant growth in human population will lead to problems with the disposal of animal waste. Livestock droppings cause some environmental problems, such as bad smells, soil eutrophication and water pollution [1, 2], but they have the potential to be used as effective biomass resources.

The black soldier fly (BSF), *Hermetia illucens* (Diptera: Stratiomyidae), is well suited for efficient utilization of livestock droppings. The larvae of *H. illucens* can feed on a wide range of organic waste, including livestock droppings, and decaying foods [3, 4]. BSF has more types and numbers of digestive enzymes than the house fly, *Musca domestica*, which means that the digestive power of BSF may be greater than that of the house fly. [5].

Rehman et al. [6] reported that when 1 kg of dairy droppings was fed to 1000 six-day-old BSF larvae, the reduction rate was 63.2% in wet weight. BSF larvae can accumulate fat and protein in their bodies [7]. The protein derived from larvae can likely be used as a substitute for soybean meal or fish meal or in animal feed [3]. Even when fed livestock droppings, BSF larvae are not only rich in proteins but also have a well-balanced amino acid profile [4, 8]. Kawasaki et al. (2019) conducted an experiment in which laying hens were fed an experimental diet, the ingredients of which were partially replaced by BSF larvae [4]. They found no adverse effects on hen weight, feed intake, or egg laying rate, while laying hens fed a diet containing BSF larvae laid eggs that were heavier and had thicker shells than those laid by control hens [4]. The fats derived from BSF larvae have been considered for use as biodiesel [9]. The properties of biodiesel derived from BSF larvae fed on food waste or livestock droppings were shown to be consistent with the European biodiesel standard EN14214 [9, 10].

Furthermore, BSF larvae convert biowaste into biomass, and the residue remaining after treatment shows compost-like properties [11]. This composting process does not emit large amounts of greenhouse gases such as methane or N₂O [12].

This BSF processing system contributes to reducing food shortage and improving the environmental health cycle. The development of the most efficient BSF rearing system requires an understanding of the associated digestive mechanism. However, the digestive mechanism of BSF has not been well elucidated. Only two types of serine proteases (HiTrypsin, HQ424575; HiChymotrypsin, HQ424574) were found and characterized in the BSF larvae [5, 13]. Given BSF’s wide range of digestive capabilities, further research into other digestive enzymes is essential. In this study, we aimed to construct an efficient BSF processing system using livestock droppings and explored the digestive enzymes involved in this process.

## Main text

### Methods

#### Insects

*H. illucens* female adults were obtained from the Fuchu campus at the Tokyo University of Agriculture and Technology, and their oviposited eggs were collected. The hatched larvae were maintained using horse, dairy cow, and laying hen droppings (Supplementary information) or an artificial diet [14] at 27 °C with a 16-h light/8-h dark cycle.

#### Feeding tests

Newly hatched larvae were fed an artificial diet for sixteen days. Then, 20 sixteen-day-old larvae were transferred to a new plastic cup. Ten grams of each livestock dropping was added to a plastic cup every three days until the larvae reached the prepupal stage. We recorded the body weight of the BSF larvae and the food intake until the end of the experiment. The feed conversion ratio (FCR) was calculated as follows:

$$\begin{gathered} FCR = \hfill \\\frac{{feed\,weight - leftovers\,weight}}{{final\,body\,weight\,of\,larvae - initial\,body\,weight\,of\,larvae}} \hfill \\ \end{gathered}$$

The feeding tests were carried out in triplicate as biological replicates.

#### Component analysis

We examined the water, crude protein and fat contents. Briefly, each sample was placed on a glass Petri dish and freeze-dried with a lyophilizer (VD-250 F, TAITECH Co., Ltd., Saitama, Japan) for 24 h. Each sample was pulverized and weighed using a Mettler balance. These dried samples were then used for analyses. The Kjeldahl method was used for crude protein analysis [15]. The Folch method was used for crude fat analysis [16].

#### RNA-seq analysis and trypsin analysis

Total RNA from the midgut and fat body samples of BSF larvae was purified with TRIzol™ reagent (Thermo Fisher Scientific Inc., Waltham, Massachusetts, USA). Then, we used a TapeStation 2200 (Agilent Technologies, Inc., Santa Clara, CA, USA) to assess RNA quality. cDNA library construction from total RNA (100 ng) was carried out using the TruSeq^®^ Stranded mRNA Library Preparation Kit (Illumina, Inc., San Diego, CA) or a NovaSeq^®^ 6000 SP Reagent Kit (Illumina, Inc., San Diego, CA, USA). These libraries (100 bp, paired-end) were sequenced using the Illumina NovaSeq 6000 or HiSeq2500 sequencer (Illumina Inc., San Diego, USA) platform.

Then, FASTQ files were assessed with the Trim Galore (v0.6.7) trimming tool (https://www.bioinformatics.babraham.ac.uk/projects/trim_galore/). Trinity software (v2.5.1) was used to construct de novo transcriptomes [17]. Transcript abundance was estimated using kallisto, and DNA sequences were translated to amino acid sequences with a transdecoder.

Gene enrichment analyses were performed using Metascape gene annotation and analysis resource 39 (https://metascape.org/). A gene list for Metascape analysis was generated from the output. The gene IDs were converted from the *H. illucens* RNA-seq data to *D. melanogaster* NCBI ID numbers with an e-value (1E-10) using the tblastx program in NCBI BLAST to construct an assignment table.

The sequences with the trypsin motif (PF00089, https://www.ebi.ac.uk/interpro/entry/pfam/PF00089/) were searched against these amino acid sequences with HMMER. Then, we annotated such sequences with BLAST KOALA (https://www.kegg.jp/blastkoala) and the tblastx program (https://blast.ncbi.nlm.nih.gov/Blast.cgi) [18, 19]. Therefore, we constructed a pipeline for choosing trypsin for *H. illucens* larvae (Fig. S1).

#### cDNA cloning

The PCR-amplified cDNA products amplified with the specific primers (Table S1) were cloned with a pIEx-1 vector (Merck, Darmstadt, Germany) and used to transform ECOS™ competent *E. coli* XL-1 blue cells (NIPPON GENE Co. Ltd., Tokyo, Japan) for subcloning. Then, nucleotide sequences were determined using a DNA analyser (3730xl, Thermo Fisher Scientific).

#### RT‒qPCR

The midgut was divided into three parts, the anterior midgut (AMG), middle midgut (MMG), and posterior midgut (PMG), according to the methods of Bonelli, M. et al. [20] (Fig. S2). Total RNA was then extracted from each part as described above.

One microgram of total RNA was treated with DNase I (Invitrogen, Van Allen Way, Carlsbad, CA, USA), and then 500 ng of DNase-treated total RNA was used as a template for cDNA synthesis using a PrimeScript™ 1st strand cDNA Synthesis Kit (Takara Co., Ltd., Tokyo, Japan) in accordance with the manufacturer’s instructions. Real-time quantitative PCR (RT‒qPCR) was performed in 20 µL reactions with 0.5 µL of cDNA template and the specific primers (Table S2) along with a KAPA SYBR Fast qRT‒PCR Kit (Nippon Genetics Co., Ltd., Tokyo, Japan) in accordance with the manufacturer’s instructions.

### Statistical analysis

The Tukey HSD method was used for determining statistical significance. Differences were considered significant at a *p* value < 0.05.

## Results and discussion

**Fig. 1:**
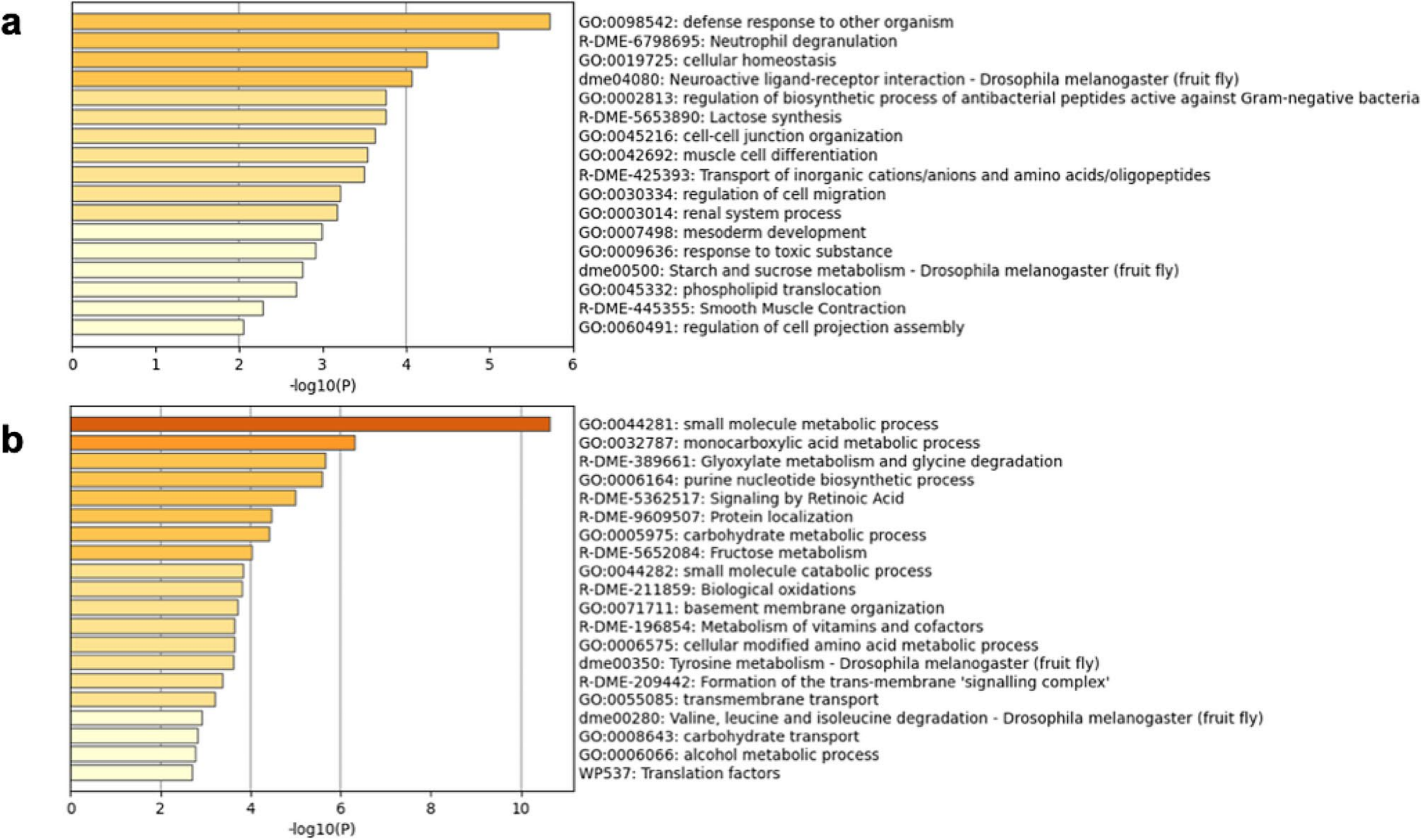
Gene enrichment analysis of the midgut and fat body. The upregulated transcripts were extracted, and gene enrichment analysis was performed. Gene enrichment analysis of fluctuating transcripts in the midgut **(a)** and fat body **(b)** using Metascape. A heatmap of enriched terms across the input transcript lists; different coloured bars indicate *p* values. The closer to dark red the colour is, the lower the *p* value

Gene enrichment analysis revealed that genes related to the Gene Ontology (GO) terms defence response to other organism (GO:0098542), neutrophil degranulation (R-DME-6,798,695), and regulation of biosynthetic process of antibacterial peptides active against gram-negative bacteria (GO:0002813) were more enriched in the midgut transcripts than in the fat body transcripts (Fig. 1-a). The GO terms in the midgut were related to immune responses to invasion by another organism, such as antimicrobial peptide production, unlike those in the fat body (Fig. 1-b). Livestock droppings also contain various types of bacteria. We speculated that BSF larvae are suitable for processing livestock droppings based on their molecular expression characteristics in the midgut.

Trypsin-like enzymes (EC3.4.21.4) play an important role in protein degradation in BSF larvae [5]. Trypsin is a type of serine protease that functions at alkaline pH and preferentially cleaves peptide bonds on the carboxyl side of arginine and lysine [21]. Trypsin activity is greater than amylase or lipase activity in the midgut of BSF larvae [13].

Insects have common digestive enzymes, and they can produce specific enzymes depending on their food [21]. Thus, we comprehensively explored digestive enzymes (Fig. S1), and we classified these transcripts into three types of proteases, namely, trypsin, cysteine protease, and aspartic acid protease, with transcripts per million (TPM) values greater than 150 (Fig. S1, Table S3). Among the three proteases, trypsin was the most abundant (Fig. S3). We extracted the trypsin transcript annotated as new brachyurin-like (TRINITY_DN59_c0_g1_i5) and determined its nucleotide sequence by cDNA cloning. The full length was 1072 bp, of which 867 bp was an open reading frame, and it was predicted to encode a polypeptide consisting of 288 amino acid residues. We named this transcript *H. illucens Brachyurin* (*HiBrachyurin*, the accession ID: LC780168).

*Brachyurin* is found in *Uca pugilator* and has been isolated mainly from marine invertebrates [22]. *Brachyurin* cleaves peptide bonds approximately 3/4 of the way from the amino terminus of collagen chains [23]. It also has broader substrate specificity than trypsin and chymotrypsin, although its cleavage efficiency against these substrates is lower [23–25].

**Fig. 2:**
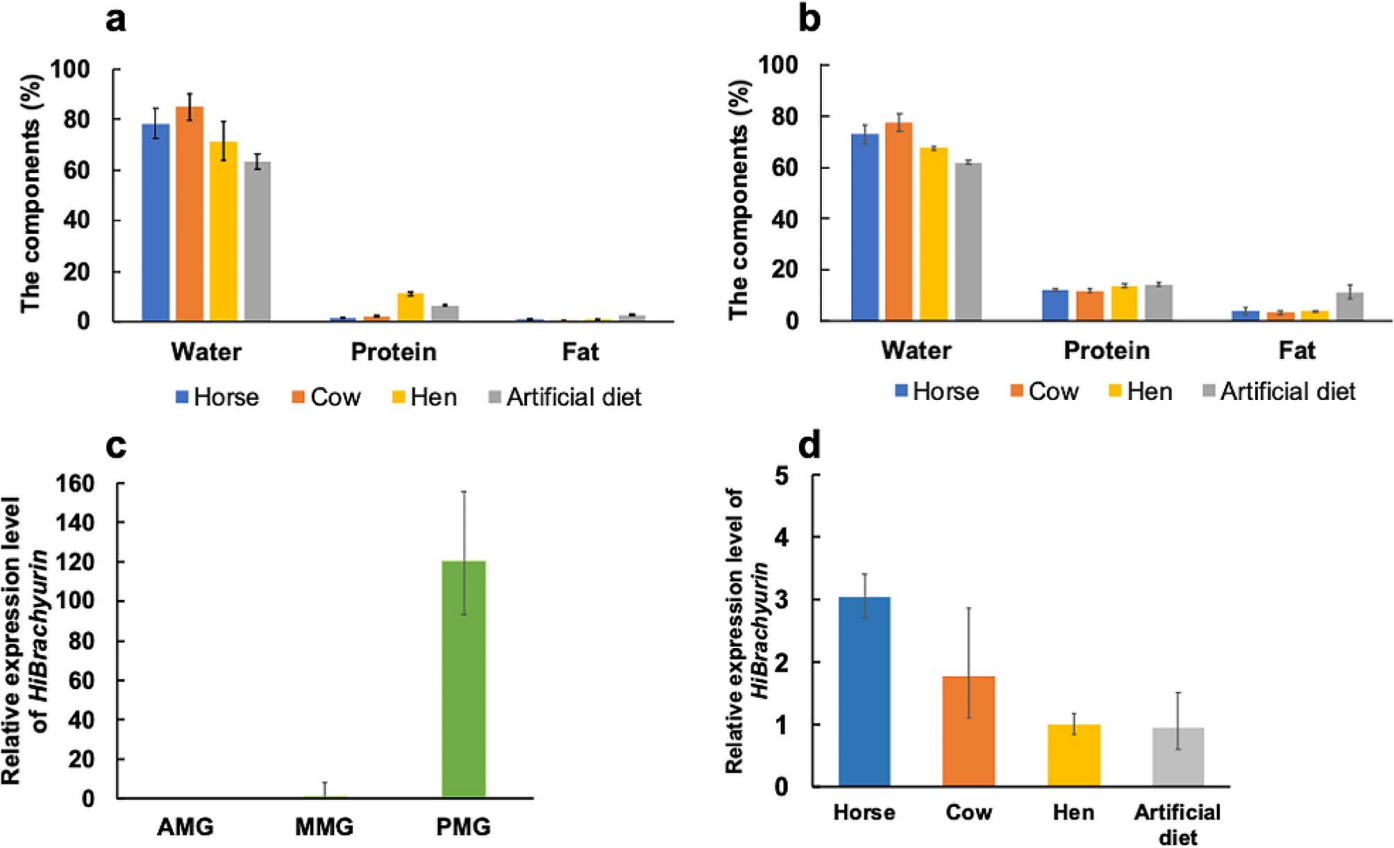
Component analysis and mRNA expression of *HiBrachyurin* in the larval midgut. Analysis of the composition of the livestock droppings **(a)** and the larvae that fed on each livestock dropping **(b)**. The livestock droppings used were from horses, cows, or laying hens. An artificial diet was used as a control. The larvae fed on each livestock dropping: horse, cow, and laying hen. Larvae that ate an artificial diet were used as a control. The y-axis indicates the component (%). The x-axis indicates water, protein, and fat. The error bars indicate the standard deviation (SD) values. mRNA expression analyses of *HiBrachyurin* in the larval midgut. **(c)** The larval midgut was divided into three parts, anterior (AMG), middle (MMG), or posterior (PMG), and each sample was processed for RT‒qPCR analysis. **(d)** The larvae were fed on droppings from horses, cows, or laying hens. The PMGs were dissected from the larvae in each group, and then each sample was processed for RT‒qPCR analysis. The relative quantification (RQ) values of the mRNA expression in the midgut of these samples were calculated. RQ values represent the relative expression levels calculated for AMG **(c)** or larval PMG fed an artificial diet **(d)** normalized to 1. Error bars represent the relative minimum/maximum expression levels of the mean RQ value (3 biological replicates). Hirs18 was used as the endogenous control

Next, we conducted a feeding test using livestock droppings to examine the efficiency of processing by BSF larvae. The larvae fed on horse droppings had the lowest feed conversion ratio (FCR) and pupated the earliest (Table 1). The survival rate was greater in the horse and cow dropping group than in the other groups (Table 1). The protein content was greater in the hen droppings than in the horse and cow droppings (Fig. 2-a); however, the protein content increased 7-fold for the larvae fed on the horse droppings, 5-fold for the larvae fed on the cow droppings, and 1.2-fold for the larvae fed on the hen droppings (Fig. 2-b, Table 1). The percentage of fat increased 4-fold for the larvae fed on the horse droppings, 7-fold for the larvae fed on the cow droppings, and 4-fold for the larvae fed on the hen droppings (Fig. 2-b, Table 1). Therefore, the most efficient method for larvae to accumulate protein and lipids in their bodies was to feed on horse droppings.

**Table 1:** The efficiency of processing livestock droppings using 16-day-old larvae

Food	Body weight (g)	Feed conversion ratio (%)	Growing period (day)	Survival rate (%)
Horse	0.074 ± 0.012^a^	8.97 ± 2.56	33 ± 6.24^a^	100 ± 0.00
Cow	0.098 ± 0.005^ab^	9.25 ± 1.90	39 ± 6.24^a^	100 ± 0.000
Hen	0.088 ± 0.004 ^ab^	16.32 ± 6.49	40 ± 9.00^a^	86.7 ± 15.28
artificial diet	0.109 ± 0.016^b^	19.43 ± 6.70	60 ± 6.24^b^	93.3 ± 2.89

All data are shown as the mean ± standard deviation (SD). The Tukey HSD method was used for determining statistical significance

Moreover, none of the zero- or ten-day-old larvae that fed on hen droppings lived for fifteen days (*n* = 100, respectively) (Table S4&5). The larvae that fed on hen droppings showed high mortality, possibly due to excess protein contained in the hen droppings being converted to ammonia [26, 27]. The survival rate of BSF larvae fed only fish or meat was significantly lower than that of larvae fed only vegetables or carbohydrates [28]. Additionally, all larvae died within 10 days when BSF larvae were fed on only fish [29]. Therefore, our results indicate the need to consider the protein content in livestock droppings to reduce BSF larval mortality.

Finally, we examined the relationships between processing livestock droppings and *HiBrachyurin*. The mRNA expression level of *HiBrachyurin* was significantly greater in the PMG than in the MMG or AMG (Fig. 2-c and S2). We also examined the mRNA expression of *HiBrachyurin* in the larval PMG fed on each livestock by RT‒qPCR and found that *HiBrachyurin* mRNA expression was greater in the larvae fed on horse droppings than in those fed on other droppings (Fig. 2-d). *HiBrachyurin* mRNA expression was consistent with the expression of proteolytic enzymes in the midgut [20]. Also, we checked whether the *Brachyurin* transcript in other insects that fed on animal droppings is expressed in the midgut. The dung beetle, *Phelotrupes auratus*, had a relatively high *Brachyurin* transcript expression in the midgut that fed on horse droppings compared to deer droppings (Figure S4). Therefore, *Brachyurin* might play a role at an early stage of protein degradation in the midgut when insects have fed on animal droppings.

## Conclusion

In summary, we identified a new type of serin protease, *HiBrachyurin*, which plays a role in larvae fed on horse droppings. BSF larvae fed on horse droppings efficiently accumulated protein and lipids in their bodies. Compatibility with the digestive enzymes of BSF larvae in the treatment of livestock droppings could be achieved to increase the efficiency of the process.

### Limitations

Our pipeline for detecting digestive enzymes makes searching for new digestive enzymes difficult without characteristic motifs.

### Electronic supplementary material

Below is the link to the electronic supplementary material.

Supplementary Material 1

## Data Availability

The RNA sequencing reads reported in this article are available in the Sequence Read Archive (SRA) under the accession IDs of the fat body groups (SRA accession numbers: DRR468816, DRR468817, and DRR468818) and the midgut groups (DRR468819, DRR468820, and DRR468821). The cloned nucleotide sequence of HiBrachyurin was submitted to the DNA Data Bank of Japan (DDBJ) under accession number LC780168.

## References

[1] Animal Products Safety Division. Food Safety and Consumer Affairs Bureau, Ministry of Agriculture, Forestry and Fisheries. 畜産環境をめぐる情勢. 2023年12月. https://www.maff.go.jp/j/chikusan/kankyo/taisaku/attach/pdf/index-169.pdf, (accessed on 11 March 2024)(In Japanese).

[2] Animal Products Safety Division. Food Safety and Consumer Affairs Bureau, Ministry of Agriculture, Forestry and Fisheries.畜産経営に起因する苦情発生状況. 2022年4月. https://www.maff.go.jp/j/chikusan/kankyo/taisaku/t_mondai/attach/pdf/archive-4.pdf, (accessed on 11 March 2024)(In Japanese).

[3] Liu C, Wang C, Yao H. Comprehensive resource utilization of waste using the black soldier fly (Hermetia illucens (L.)) (diptera: Stratiomyidae). Animals. 2019;9. 10.3390/ani9060349. 10.3390/ani9060349 PMC6616655 31200503

[4] Kawasaki K, Hashimoto Y, Hori A, Kawasaki T, Hirayasu H, Iwase SI, et al. Evaluation of black soldier fly (*Hermetia illucens*) larvae and pre-pupae raised on household organic waste, as potential ingredients for poultry feed. Animals. 2019;9(3). 10.3390/ani9030098. 10.3390/ani9030098 PMC6466380 30893879

[5] Kim W, Bae S, Kim A, Park K, Lee S, Choi Y (2011). Characterization of the molecular features and expression patterns of two serine proteases in *Hermetia illucens* (Diptera: Stratiomyidae) larvae. BMB Rep.

[6] Rehman K, ur, Rehman A, Cai M, Zheng L, Xiao X, Somroo AA (2017). Conversion of mixtures of dairy manure and soybean curd residue by black soldier fly larvae (*Hermetia illucens* L). J Clean Prod.

[7] Nyakeri EM, Ogola HJO, Ayieko MA, Amimo FA (2017). Valorisation of organic waste material: growth performance of wild black soldier fly larvae (*Hermetia illucens*) reared on different organic wastes. J Insects as Food Feed.

[8] Barragan-Fonseca KB, Dicke M, van Loon JJA (2017). Nutritional value of the black soldier fly (*Hermetia illucens* L.) and its suitability as animal feed - a review. J Insects as Food Feed.

[9] Li Q, Zheng L, Cai H, Garza E, Yu Z, Zhou S (2011). From organic waste to biodiesel: black soldier fly, *Hermetia illucens*, makes it feasible. Fuel.

[10] Zheng L, Li Q, Zhang J, Yu Z (2012). Double the biodiesel yield: rearing black soldier fly larvae, Hermetia illucens, on solid residual fraction of restaurant waste after grease extraction for biodiesel production. Renewable Energy.

[11] Gold M, Tomberlin JK, Diener S, Zurbrügg C, Mathys A. (2018, December 1). Decomposition of biowaste macronutrients, microbes, and chemicals in black soldier fly larval treatment: A review. Waste Management, Vol. 82, pp. 302–31810.1016/j.wasman.2018.10.022. 10.1016/j.wasman.2018.10.022 30509593

[12] Parodi A, Gerrits WJJ, Van Loon JJA, De Boer IJM, Aarnink AJA, Van Zanten HHE (2021). Black soldier fly reared on pig manure: Bioconversion efficiencies, nutrients in the residual material, greenhouse gas and ammonia emissions. Waste Manag.

[13] Kim W, Bae S, Park K, Lee S, Choi Y, Han S, Koh Y (2011). Biochemical characterization of digestive enzymes in the black soldier fly, *Hermetia illucens* (Diptera: Stratiomyidae). J Asia Pac Entomol.

[14] Nakagawa A, Sakamoto T, Kanost MR, Tabunoki H (2023). The development of New methods to stimulate the production of antimicrobial peptides in the Larvae of the black soldier fly *Hermetia illucens*. Int J Mol Sci.

[15] Kjeldahl J (1883). Neue Methode Zur Bestimmung Des Stickstoffs in Organischen Körpern. Fresenius Z f anal Chemie.

[16] Folch J, Lees M, Sloane Stanley GH (1957). A simple method for the isolation and purification of total lipides from animal tissues. J Biol Chem.

[17] Grabherr MG, Haas BJ, Yassour M, Levin JZ, Thompson DA, Amit I (2011). Full-length transcriptome assembly from RNA-Seq data without a reference genome. Nat Biotechnol.

[18] Kanehisa M, Sato Y, Kawashima M, Furumichi M, Tanabe M (2016). KEGG as a reference resource for gene and protein annotation. Nucleic Acids Res.

[19] Salgaonkar BB, Kabilan M, Braganca JM (2011). Basic local alignment search tool. World J Microbiol Biotechnol.

[20] Bonelli M, Bruno D, Caccia S, Sgambetterra G, Cappellozza S, Jucker C (2019). Structural and functional characterization of hermetia illucens larval midgut. Front Physiol.

[21] Terra WR, Ferreira C (1994). Insect digestive enzymes: properties, compartmentalization and function. Comp Biochem Physiol -- Part B Biochem.

[22] Kristjánsdóttir S, Gudmundsdóttir Á (2000). Propeptide dependent activation of the Antarctic krill euphauserase precursor produced in yeast. Eur J Biochem.

[23] Tsu CA, Craik CS (1996). Substrate recognition by recombinant serine collagenase 1 from Uca pugilator. J Biol Chem.

[24] Lin YB, Rong JJ, Wei XF, Sui ZX, Xiao J, Huang DW (2021). Proteomics and ultrastructural analysis of Hermetia illucens (Diptera: Stratiomyidae) larval peritrophic matrix. Proteome Sci.

[25] Malawey AS, Mercati D, Love CC, Tomberlin JK (2019). Adult Reproductive Tract Morphology and Spermatogenesis in the black soldier fly (Diptera: Stratiomyidae). Ann Entomol Soc Am.

[26] Cammack JA, Tomberlin JK. The impact of diet protein and carbohydrate on select life-history traits of the black soldier fly Hermetia illucens (L.) (Diptera: Stratiomyidae). Insects. 2017;8(2). 10.3390/insects8020056. 10.3390/insects8020056 PMC5492070 28561763

[27] Dias ACA, Rodrigues MMS, Silva AA (2019). Effect of acute and chronic exposure to ammonia on different larval instars of Anopheles darlingi (Diptera: Culicidae). J Vector Ecol.

[28] Meneguz M, Schiavone A, Gai F, Dama A, Lussiana C, Renna M, Gasco L (2018). Effect of rearing substrate on growth performance, waste reduction efficiency and chemical composition of black soldier fly (Hermetia illucens) larvae. J Sci Food Agric.

[29] Hirayasu H, Seyama T, Wachi N, Yoshida G, Kasai K, Fujitani Y (2017). Food Waste Suitable for Treatment Using Black Soldier Fly (Hermetia illucens) Larvae. Bull Res Inst Env Agr Fish Osaka.

