# Supplementary material for: High expression of serine protease, Brachyurin in the posterior midgut of black soldier fly (Hermetia illucens) during horse dropping processing
Source: BMC Res Notes. 2024 Jun 29;17:182. doi: 10.1186/s13104-024-06846-0 (PMC11218125; doi:10.1186/s13104-024-06846-0)
Supplement: Supplementary file 1 — Supplementary Material 1 [file 13104_2024_6846_MOESM1_ESM.pdf]

High Expression of Serine Protease, *Brachyurin* in the Posterior Midgut of Black Soldier Fly (*Hermetia illucens*) During Horse Dropping Processing

Megumi Wakuda[1 †], Takuma Sakamoto[1†], Akane Tanaka[2,3], Satoshi Sugimura[4], Yuki Higashiura[1], Takeru Nakazato[5], Hidemasa Bono[5,6,7] and Hiroko Tabunoki[1, 3*]

[1] Department of Science of Biological Production, Graduate School of Agriculture, Tokyo University of Agriculture and Technology, Fuchu, Tokyo, Japan

[2] Division of Animal Life Science, Institute of Agriculture, Tokyo, Japan; Laboratory of Comparative Animal Medicine, Division of Animal Life Science, Institute of Agriculture, Tokyo University of Agriculture and Technology, Fuchu, Tokyo, Japan.

[3] Cooperative Major in Advanced Health Science, Graduate School of Bio-Applications and System Engineering, Tokyo University of Agriculture and Technology, Fuchu, Tokyo, 183-8509, Japan

[4] Institute of Global Innovation Research, Tokyo University of Agriculture and Technology, 3-5-8 Saiwai-cho, Fuchu, Tokyo, 183-8509, Japan.

[5] Database Center for Life Science (DBCLS), Joint Support-Center for Data Science Research, Research Organization of Information and Systems (ROIS), Mishima, Shizuoka, Japan

[6]Laboratory of Bio-DX, Genome Editing Innovation Center, Hiroshima University, 3-10-23 Kagamiyama, Hi-gashi-Hiroshima city, Hiroshima, 739-0046, Japan

[7]Laboratory of Genome Informatics, Graduate School of Integrated Sciences for Life, Hiroshima University, 3-10-23 Kagamiyama, Higashi-Hiroshima city, Hiroshima, 739-0046, Japan

***Corresponding author**

Hiroko Tabunoki

Tel & Fax: +81-42-367-5613

E-mail: h_tabuno@cc.tuat.ac.jp

†Megumi Wakuda and Takuma Sakamoto contributed equally to this work.

Materials and Methods

Livestock droppings

We used three kinds of livestock droppings; horse, dairy cow, and laying hen. All of these droppings were collected from each livestock farm on the Fuchu campus. The horse was raised on timothy hay (*Phragmites australis*), alfalfa hay, rock salt, carrot, apple, and horse feed (Havens co. ltd., Vierlingsbeek, Netherlands).

The dairy cow was fed on dent corn, Sudan grass, alfalfa hay, defatted soybeans (Shimizu shiryo co., ltd., Hachiouji, Tokyo, Japan), concentrated feed (Morinaga rakunou co., Ltd., Minato-ku, Tokyo, Japan), and vitamins (Alltech, Fukuoka city, Fukuoka, Japan).

Adult commercial laying hens (White Leghorn) aged 328 to 354 days were used. The hens were housed in individual cages measuring 25 cm × 40 cm × 43 cm (width × depth × height). Room temperature was maintained at 25.0°C. Lighting was provided by fluorescent bulbs set at an intensity of 500 lux at the feeder. The light cycle was 14 hours light and 10 hours dark, with the lights on from 06:00 to 20:00. Hens had ad libitum access to water and feed. Commercial diets for laying hens were used. The droppings were collected and weighed. These hen droppings were then processed for compositional analysis and used as BSF larval diets.

Feeding tests

Newly hatched larvae were fed an artificial diet for ten-days. Then, 20 ten-day-old larvae were transferred to a new plastic cup. Ten grams of each livestock droppings was added to a plastic cup every three days until the larvae reached the prepupal stage. We recorded the body weight of the BSF larvae, and the food intake until the end of the experiment. The feed conversion ratio (FCR) was calculated as follows:

$$FCR = \frac{feed\ weight - leftovers\ weight}{final\ body\ weight\ of\ larvae - \ initial\ body\ weight\ of\ larvae}$$

Also, we performed feeding tests against newly hatched larvae (zero-day-old larvae). The feeding tests were carried out in triplicate as biological replicates.

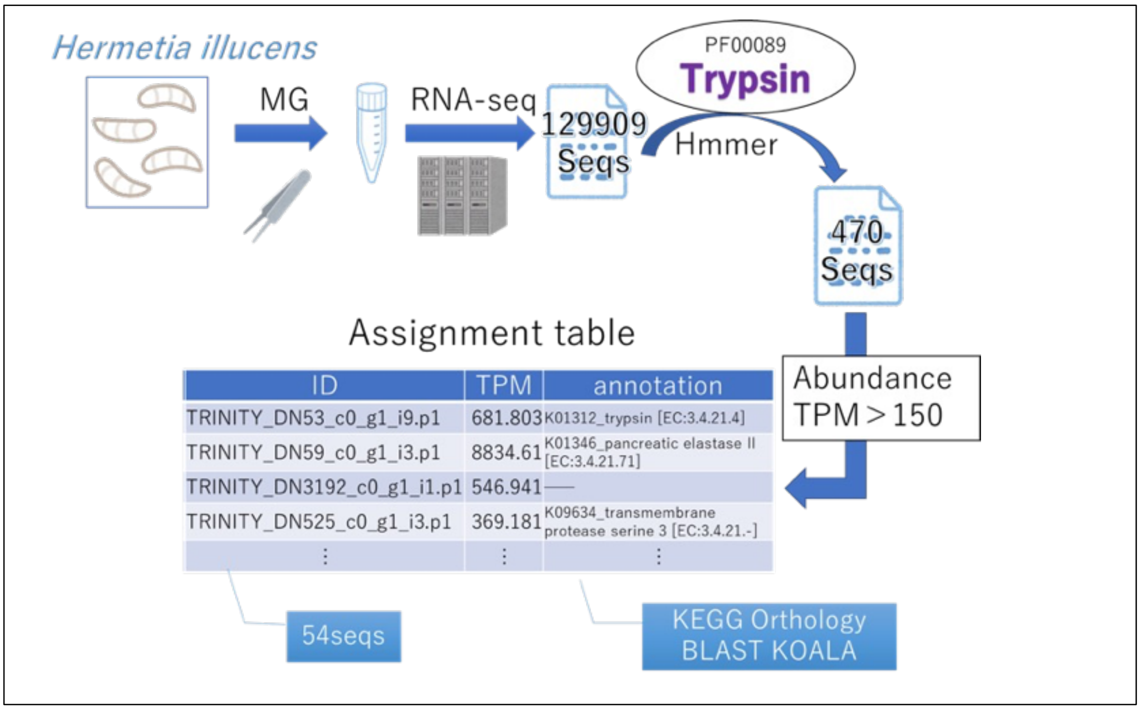

Fig. S1 A pipeline for trypsin selection in *H. illucens* larvae. We constructed digestive enzymes extract pipeline using a public database, then extracted global proteases from the midgut transcriptome of *H. illucens* larvae. 129,909 sequences were found, and then 470 sequences were extracted by HMM search using the trypsin motif. These transcripts were cut-off with TPM value below 150. Finally, 54 sequences were obtained and annotated using KEGG orthology and BLAST KOARA database.

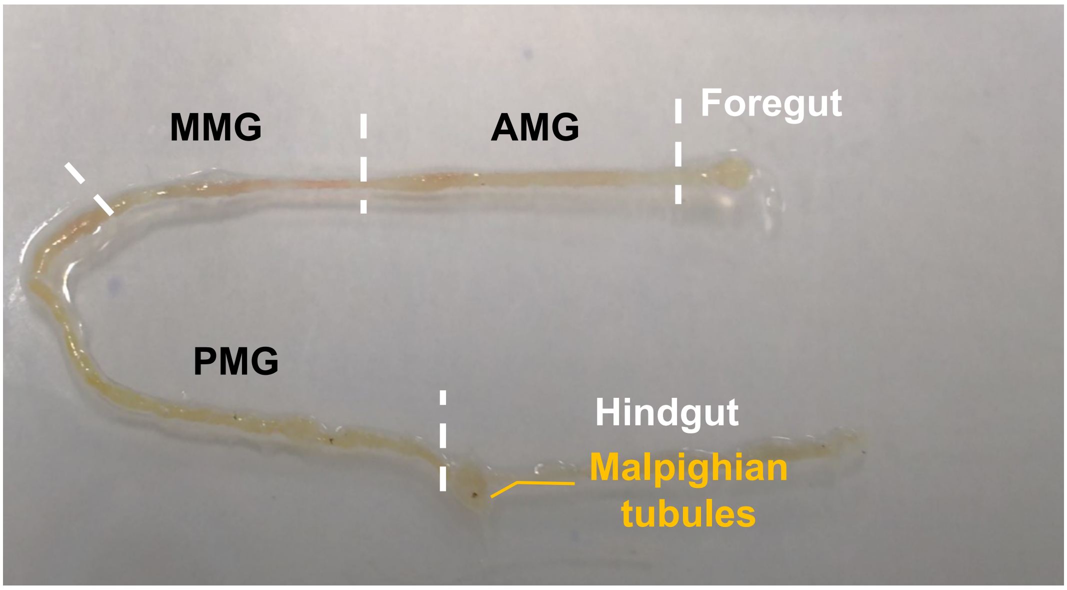

Fig. S2 The midgut of the final instar larva in *H. illusens*. The larval midgut was divided into three parts; anterior midgut (AMG), middle midgut (MMG), posterior midgut (PMG) according to the methods of Bonelli et al. (2019).

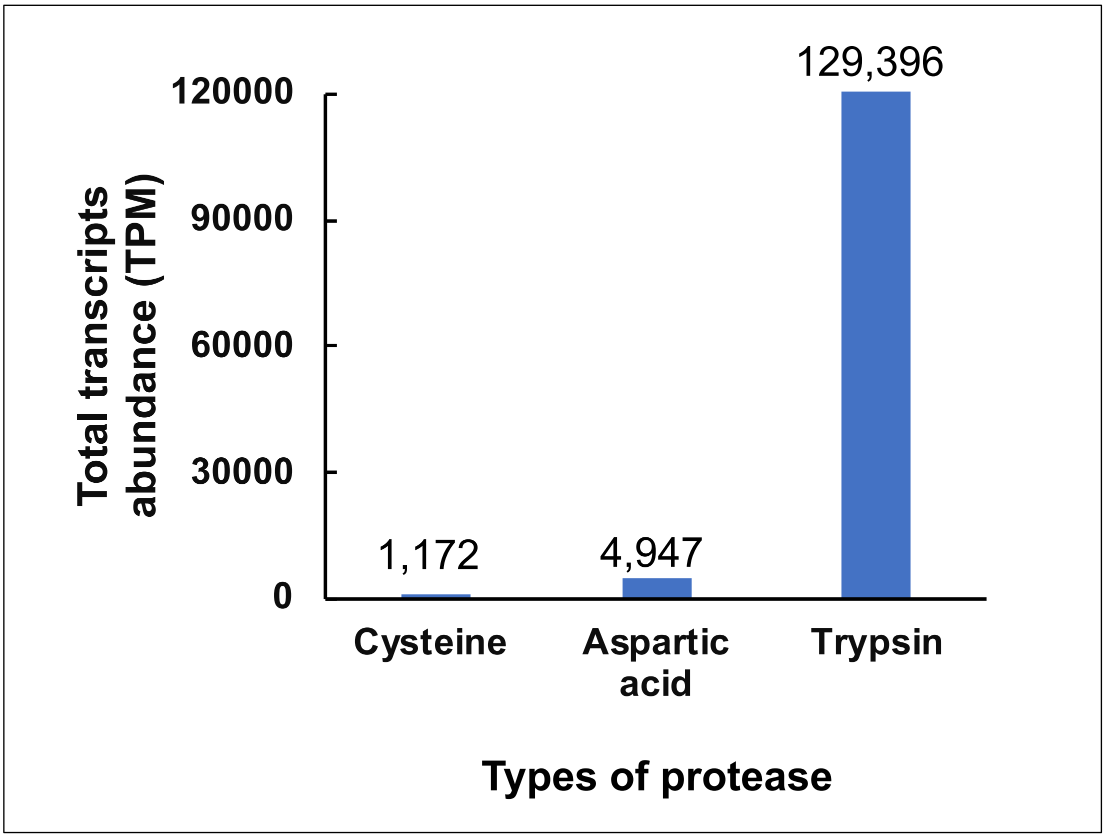

Fig. S3 Annotation for three types of proteases, and their expression. The y-axis indicates the average transcripts per kilobase million (TPM) values for midgut transcripts assigned to proteases. The x-axis indicates the type of protease.

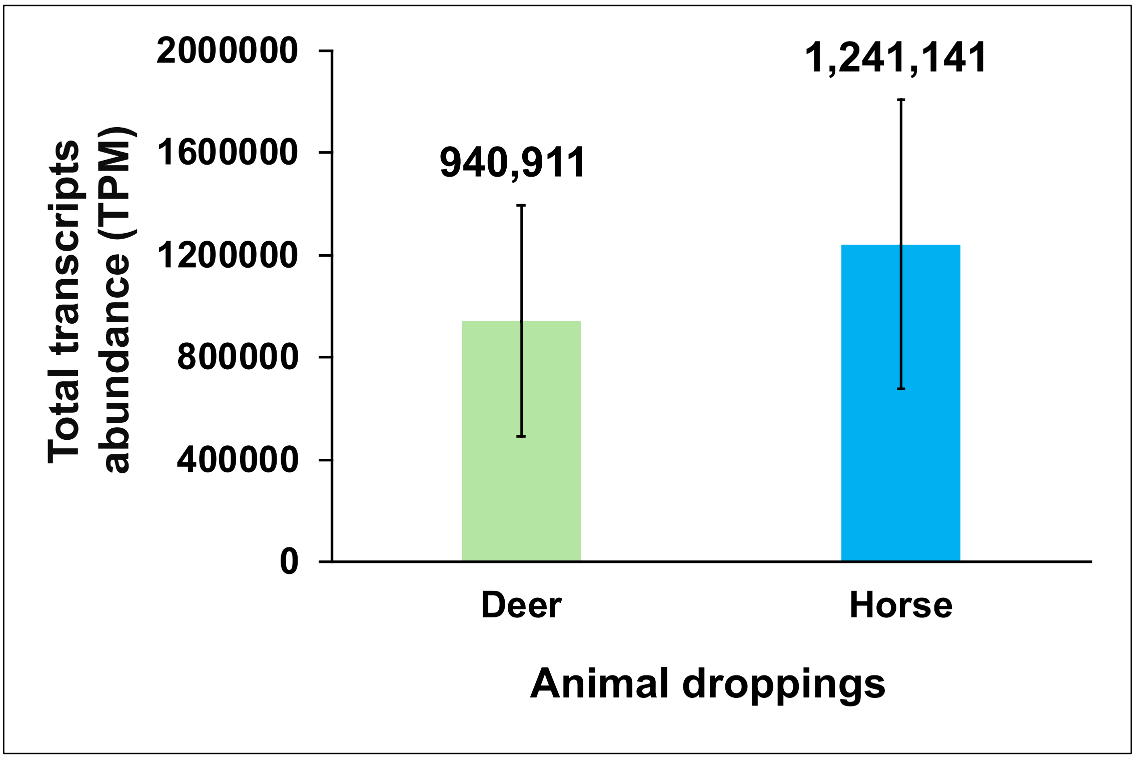

Fig. S4 The *Brachyurin* transcript expression in the dung beetle's midgut, *Phelotrupes auratus*.

The RNA-Seq data on six midgut samples that fed on deer droppings (the Sequence Read Archive accession numbers: DRR357601, DRR357602, DRR357603, DRR357607, DRR357608, and DRR357609), six midgut samples that fed on horse droppings (DRR357589, DRR357590, DRR357589, DRR357592, DRR357593, and DRR357594) were used. The *Brachyurin* transcript was extracted using the tblastx method according to Sakamoto T. et al. (BMC Genomics 2022, 23:751). The y-axis indicates the average transcripts per kilobase million (TPM) values for midgut transcripts assigned to *Brachyurin*. The x-axis indicates each animal's droppings.

Tables

Table S1. Primers for cDNA cloning were used in this study.

Gene name	Forward (5'-3')	Reverse (5'-3')
HiBrachyuran	ATAGGATCCCATGAAATCGTTCGCA	CGTAAGCTTCGTAATTTAGTCGCGAAG
Insert check	GAACGCCAGCACATGGAC	CCGGAAGACCCCGAGGAT

Table S2. Primers for RT-qPCR were used in this study.

	Forward(5’-3’)	Reverse(5’-3’)
HiBrachyuran	GGTGACTCAGGTGGTCCATT	AAGATTGTGTTCCTACAGTGGAAAT
18S rRNA	CGATGGCAAGTACACACAGC	AATCCACCAACGCGACATTG

Table S3. Protease transcripts with TPM value higher than 150.

ID	TPM	Annotation
TRINITY_DN10023_c0_g1_i1	32429.8	chymotrypsin
TRINITY_DN3327_c0_g1_i1	18438.9	Trypsin-delta like
TRINITY_DN59_c0_g1_i3	8834.61	brachyurin-like
TRINITY_DN790_c0_g1_i7	5378.09	trypsin [EC:3.4.21.4]
TRINITY_DN53_c0_g1_i2	4993.17	trypsin [EC:3.4.21.4]
TRINITY_DN59_c0_g1_i5	4749.23	brachyurin-like
TRINITY_DN790_c0_g1_i19	3261.87	trypsin [EC:3.4.21.4]
TRINITY_DN1749_c0_g1_i1	3125.09	brachyurin-like
TRINITY_DN4827_c0_g1_i12	3027.74	chymotrypsin BI-like
TRINITY_DN59_c0_g1_i2	3004.95	brachyurin-like
TRINITY_DN4827_c0_g1_i11	2835.76	brachyurin-like
TRINITY_DN4827_c0_g1_i9	2641.28	chymotrypsin BI-like
TRINITY_DN790_c0_g1_i14	2301.28	trypsin [EC:3.4.21.4]
TRINITY_DN53_c0_g1_i11	2192.57	trypsin [EC:3.4.21.4]
TRINITY_DN7758_c0_g1_i2	1692.55	trypsin [EC:3.4.21.4]
TRINITY_DN790_c0_g1_i3	1641.48	Trypsin-delta like
TRINITY_DN790_c0_g1_i4	1550.34	trypsin [EC:3.4.21.4]
TRINITY_DN790_c0_g1_i15	1523.7	trypsin [EC:3.4.21.4]
TRINITY_DN1009_c1_g1_i1	1484.16	Trypsin-delta like
TRINITY_DN10023_c0_g1_i2	1462.29	trypsin [EC:3.4.21.4]
TRINITY_DN790_c0_g1_i20	1306.52	trypsin [EC:3.4.21.4]
TRINITY_DN790_c0_g1_i18	1233.99	trypsin [EC:3.4.21.4]
TRINITY_DN53_c0_g1_i12	1074.18	trypsin [EC:3.4.21.4]
TRINITY_DN4827_c0_g1_i3	1032.69	chymotrypsin BI-like
TRINITY_DN53_c0_g1_i6	1020.39	serine protease SP24-like
TRINITY_DN790_c0_g1_i10	1000.99	trypsin [EC:3.4.21.4]
TRINITY_DN3666_c0_g1_i2	901.848	brachyurin-like
TRINITY_DN1009_c1_g1_i3	859.005	Trypsin-delta like
TRINITY_DN6456_c0_g1_i2	849.79	trypsin-like protease
TRINITY_DN790_c0_g1_i12	849.308	trypsin [EC:3.4.21.4]
TRINITY_DN4827_c0_g1_i8	719.3	chymotrypsin BI-like
TRINITY_DN53_c0_g1_i9	681.803	trypsin [EC:3.4.21.4]
TRINITY_DN6611_c0_g1_i3	668.665	serine protease SP24-like
TRINITY_DN3192_c0_g1_i1	546.941	trypsin [EC:3.4.21.4]
TRINITY_DN59_c0_g1_i7	479.812	brachyurin-like
TRINITY_DN1009_c1_g1_i2	428.515	trypsin [EC:3.4.21.4]
TRINITY_DN1413_c1_g1_i1	423.46	Trypsin-delta like
TRINITY_DN10023_c0_g1_i3	414.541	chymotrypsin
TRINITY_DN1069_c0_g1_i1	390.48	trypsin [EC:3.4.21.4]
TRINITY_DN525_c0_g1_i3	369.181	collagenase-like
TRINITY_DN53_c0_g1_i8	355.376	serine protease SP24-like
TRINITY_DN6203_c0_g1_i3	345.005	trypsin-like protease
TRINITY_DN790_c0_g1_i16	324.375	trypsin [EC:3.4.21.4]
TRINITY_DN9449_c0_g1_i3	289.198	trypsin [EC:3.4.21.4]
TRINITY_DN66295_c0_g1_i1	280.885	trypsin [EC:3.4.21.4]
TRINITY_DN9449_c0_g1_i1	257.179	trypsin [EC:3.4.21.4]
TRINITY_DN59_c0_g1_i1	254.388	brachyurin-like
TRINITY_DN230_c0_g1_i1	231.159	trypsin 3A1-like
TRINITY_DN790_c0_g1_i8	211.522	trypsin [EC:3.4.21.4]
TRINITY_DN790_c0_g1_i17	209.76	trypsin [EC:3.4.21.4]
TRINITY_DN230_c0_g1_i2	208.714	trypsin 3A1-like
TRINITY_DN3064_c0_g1_i1	187.452	mucin-2-like
TRINITY_DN1545_c0_g1_i1	183.846	mite allergen Eur m 3-like
TRINITY_DN525_c0_g1_i2	169.362	collagenase-like

Table S4. The efficiency of processing livestock droppings using 0-day-old larvae.

Food	Body weight (g)	Growing period (day)	Survival rate (%)
horse	0.098±0.016	50.3±11.72	100±0.00
cow	0.113±0.009	42.0±8.66	100±0.00
hen	N.D	N.D	0

Newly hatched larvae (n=100, respectively) fed an each livestock droppings until prepupal stage. Ten grams of each livestock droppings was added to a plastic cup every three days until the larvae reached the prepupal stage. We recorded the body weight of the BSF larvae until the end of the experiment. The feeding tests were carried out in triplicates as biological replication. N.D mean not detect. All data are shown as the mean ± standard deviation (SD). The Tukey HSD method was used for determining statistical significance.

Table S5. The efficiency of processing livestock droppings using 10-day-old larvae.

Food	Body weight (g)	Feed conversion rate(%)	Growing period (day)	Survival rate (%)
horse	0.077±0.031[a]	7.05±1.40	34±3.00	96.7±2.89
cow	0.101±0.032[b]	6.09±1.83	36±4.58	96.7±.5.77
hen	N.D	N.D	N.D	0

Newly hatched larvae were fed an artificial diet for ten-days. Then, 20 ten-days-old larvae were transferred to a new plastic cup. Ten grams of each livestock droppings was added to a plastic cup every three days until the larvae reached the prepupal stage. We recorded the body weight of the BSF larvae and the food intake until the end of the experiment. N.D mean not detect. All data are shown as the mean ± standard deviation (SD). The Tukey HSD method was used for determining statistical significance.
